# Artificial Intelligence in Pediatric Cardiovascular Genetics: From Multimodal Diagnosis to Risk Prediction and Precision Therapeutics

**DOI:** 10.3390/ijms27188284

**Published:** 2026-09-17

**Authors:** Nikola Ilić, Staša Krasić, Vladislav Vukomanović, Adrijan Sarajlija

**Affiliations:** 1 Clinical Genetics Outpatient Clinic, Mother and Child Health Care Institute of Serbia “Dr Vukan Cupic”, 11070 Belgrade, Serbia; adrijan.sarajlija@imd.org.rs; 2 Department of Cardiology, Mother and Child Health Care Institute of Serbia “Dr Vukan Cupic”, 11070 Belgrade, Serbia; stasa.krasic@imd.org.rs (S.K.); vladislav.vukomanovic@imd.org.rs (V.V.); 3 Department of Pediatrics, Faculty of Medicine, University of Belgrade, 11000 Belgrade, Serbia

**Keywords:** artificial intelligence, pediatric cardiovascular genetics, inherited cardiovascular disease, genomic medicine, cardiomyopathy, channelopathy, congenital heart disease, risk stratification, precision medicine

## Abstract

Artificial intelligence (AI) is increasingly being explored as a bridge between high-dimensional cardiovascular phenotyping and genomic interpretation in children with inherited and congenital heart disease. This review focuses specifically on where AI-assisted approaches in pediatric cardiovascular genetics have progressed beyond proof-of-concept, how the maturity of evidence differs between general genomic tools and pediatric cardiovascular applications, and what is required for responsible clinical translation. Inherited and rare cardiovascular disorders are characterized by genetic heterogeneity, incomplete penetrance, variable expressivity, and age-dependent phenotypes, while modern sequencing, imaging, and longitudinal monitoring generate data that are difficult to integrate using conventional approaches alone. AI-assisted methods are being investigated across diagnostic, prognostic, and therapeutic domains, including computational phenotyping, genomic variant prioritization, electrocardiographic and imaging analysis, multimodal risk prediction, and selection of candidates for precision therapies. The strongest pediatric cardiovascular evidence currently comes from externally evaluated ECG and echocardiographic models and from large outcome-prediction cohorts, whereas many genomic tools—including variant callers and pathogenicity predictors—remain general-purpose methods that have not been specifically validated in pediatric cardiovascular genetics. Recent disease-specific genomic models and automated reanalysis frameworks illustrate a shift toward more clinically contextualized interpretation, but current evidence remains limited by retrospective design, small or selectively assembled rare-disease cohorts, domain shift, population bias, incomplete calibration, and uncertain clinical utility. Accordingly, AI should be viewed as a governed decision-support layer rather than an autonomous authority. With external validation, transparent reporting, longitudinal monitoring, and multidisciplinary oversight, AI has the potential to augment specialist expertise and may support earlier diagnosis, improved risk stratification, and more individualized treatment.

## 1. Introduction

Rare and inherited cardiovascular diseases represent a clinically and genetically heterogeneous group of disorders that may become apparent at any stage of childhood, from the prenatal period and neonatal intensive care setting to adolescence. This spectrum includes inherited cardiomyopathies, cardiac channelopathies, heritable aortopathies, familial dyslipidemias, congenital heart defects, and multisystem genetic or metabolic disorders with cardiovascular involvement. Although each individual condition is uncommon, their collective burden is substantial. Many are associated with progressive heart failure, malignant arrhythmias, sudden cardiac death, or a lifelong need for specialized surveillance and treatment. Early identification is therefore essential not only for the affected child but also for relatives who may carry the same disease-associated variant while remaining clinically asymptomatic [1,2].

The expanding availability of next-generation sequencing has transformed the investigation of pediatric cardiovascular disorders. Targeted gene panels, exome sequencing, genome sequencing, and, increasingly, transcriptomic and other multi-omics approaches can identify molecular diagnoses that would previously have remained unresolved. Nevertheless, the interpretation of genomic data remains a major bottleneck. Each patient may harbor thousands of rare variants, while the clinical relevance of many detected variants remains uncertain. Accurate interpretation requires the integration of allele frequency, predicted molecular consequence, gene–disease validity, inheritance pattern, segregation data, functional evidence, and detailed phenotypic information. This process becomes particularly difficult when the phenotype is incomplete, when multiple potentially relevant variants are identified, or when the causal alteration lies outside well-characterized coding regions [1].

Previous reviews have mapped AI broadly across pediatric cardiology, including diagnosis, imaging, interventions, prognosis, omics, precision medicine, and fetal cardiology, while emphasizing that most published systems remained in silico and rarely demonstrated deployable clinical value [3]. The present review addresses a narrower intersection: AI-assisted pediatric cardiovascular genetics, with particular emphasis on genomic interpretation, multimodal genotype–phenotype integration, and the evidentiary and governance requirements for clinical translation. This distinction is important because many widely cited computational tools were developed for general genomics or adult cardiovascular medicine and have not been specifically validated in pediatric cardiovascular genetics.

In this review, AI is used as an umbrella term encompassing machine learning, deep learning, natural-language processing, foundation models, and selected data-driven computational approaches. These methods differ substantially in architecture and intended use: some perform narrow classification or prediction tasks, whereas others prioritize variants, extract phenotypes, or integrate multiple data modalities. Throughout the review, we therefore distinguish general genomic methods from evidence generated directly in pediatric cardiovascular populations and avoid treating all computational approaches as equivalent forms of clinical AI.

Despite rapid progress, pediatric cardiovascular genetics remains a difficult environment for model development. Compared with adult cardiovascular medicine, available datasets are smaller, more fragmented, and more heterogeneous, while rare disorders are especially vulnerable to class imbalance, familial clustering, overfitting, and poor representation of diverse ancestral and socioeconomic populations. Additional concerns include interpretability, inconsistent data standards, protection of sensitive genomic information, regulatory oversight, and responsibility for AI-assisted decisions. High retrospective performance alone is therefore insufficient to establish clinical utility [1,2].

**Figure 1:**
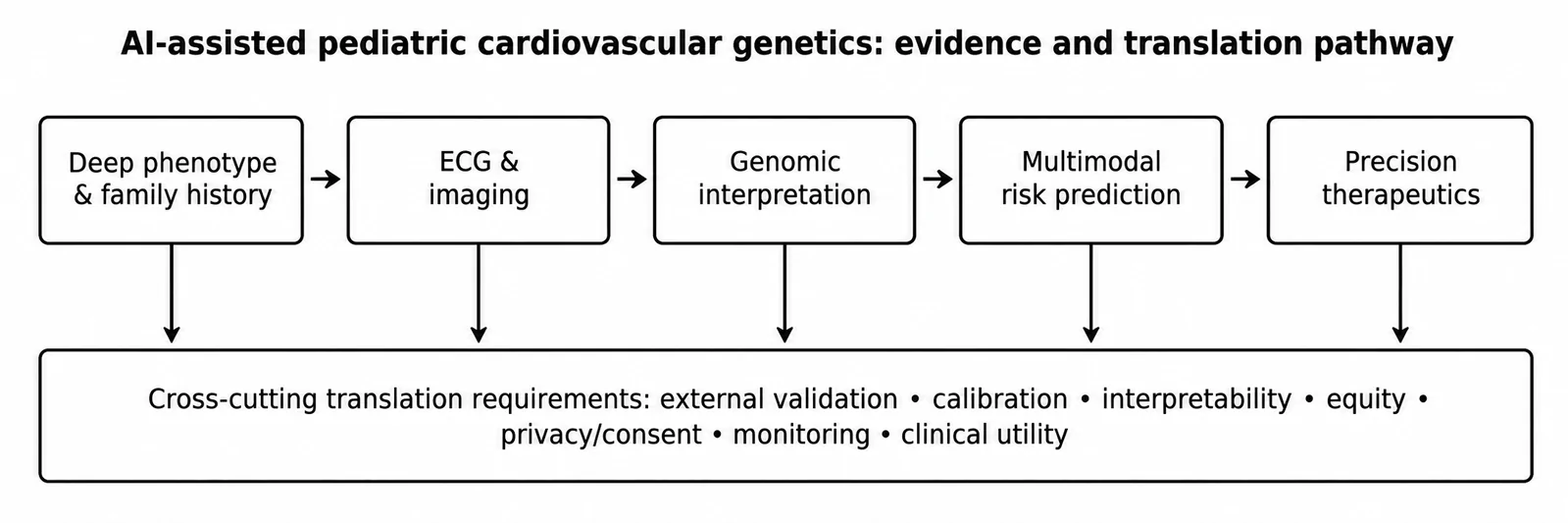
Organizing framework for AI-assisted pediatric cardiovascular genetics, linking phenotype, cardiovascular data, genomic interpretation, risk prediction, precision therapeutics, and cross-cutting requirements for clinical translation.

This review asks three linked questions: (1) Which AI-assisted applications in pediatric cardiovascular genetics have moved beyond proof-of-concept toward external or clinically meaningful validation? (2) Where does the evidence derive from pediatric cardiovascular populations rather than from general genomics or adjacent areas of cardiology? (3) What methodological, ethical, and governance requirements must be met before such systems can support routine care? We organize the evidence around diagnostic and genomic applications, disease-specific and prognostic use cases, precision therapeutics, and clinical translation, with the genomics-specific synthesis and the translation framework representing the principal areas of emphasis (Figure 1).

For this narrative review, literature was identified through targeted searches of PubMed and major journal/publisher databases through 31 August 2026, supplemented by reference chaining from relevant reviews and consensus documents. Priority was given to primary studies with pediatric populations, independent or temporal validation, cardiovascular–genetic relevance, or clinically informative methodological limitations, together with recent genomic and AI-translation frameworks. Because the review is narrative rather than systematic, the search was intended to provide representative and clinically informative evidence rather than an exhaustive quantitative synthesis (Table 1).

**Table 1:** Representative primary studies illustrating the current evidence base for AI-assisted pediatric cardiovascular and genomic applications.

Interpretation for This Review	Validation and Principal Result	AI-Assisted Task	Population	Study/Domain
Large pediatric external validation; demonstrates diagnostic signal from ECG but not genetics-specific integration.	AUROC 0.915 internally and 0.917/0.907 in two external cohorts	Deep-learning CHD detection from pediatric ECG	65,869 development cases; 12,000 internal test cases; external cohorts of 7137 and 8121	Chen et al. [4]—pediatric ECG/CHD
Direct evidence of domain shift and the importance of acquisition, labeling, and population composition.	Critical-CHD AUROC 0.94 internally, 0.77 externally, improving to 0.87 after retraining	EchoFocus-CHD detection of critical and non-critical CHD	54,727 internal echocardiograms and 3356 external echocardiograms	Lukyanenko et al. [5]—echocardiography/CHD
Cardiovascular-specific genomic AI; promising for variant interpretation but not yet a pediatric clinical workflow.	Improved held-out rare-variant prediction with replication in ClinVar annotations	Disease-specific fine-tuning of genomic foundation models (DYNA)	Rare coding variants in inherited cardiovascular disease and regulatory/splicing variant datasets	Zhan et al. [6]—cardiovascular genomics
Strong evidence for scalable genomic reanalysis, although not cardiovascular-specific.	Recovered 90% of known diagnoses; identified 241 diagnoses (5.1%) in the undiagnosed cohort	Automated inheritance-aware variant prioritization and iterative reanalysis (Talos)	1089 individuals for validation; 4735 undiagnosed individuals for scaled reanalysis	Welland et al. [7]—rare-disease genomics
Large cohort and temporal split; illustrates strong discrimination but also the need to exclude downstream variables that create temporal leakage.	Reported test-set AUCs approximately 0.89–0.97	ML prediction of four major postoperative adverse outcomes	23,000 consecutive pediatric surgical patients; temporal train/test split	Tong et al. [8]—congenital heart surgery
Multicenter longitudinal evidence; clinically relevant but derived from a specific high-risk CHD population.	Time-dependent AUC up to 0.838 using peri-Stage-1 data	ML prediction of 5-year transplant-free survival	549 infants from 15 centers in the Pediatric Heart Network SVR program	Smith et al. [9]—HLHS prognosis
Shows that complex algorithms do not necessarily yield large clinical gains when outcome determinants are incompletely captured.	Random survival forest concordance index 0.68, modestly above Cox modeling	Machine-learning survival modeling	4150 pediatric heart-transplant recipients	Ashfaq et al. [10]—pediatric transplantation
One of the strongest multicenter pediatric/CHD validation examples, but not specifically a cardiovascular-genetics model.	AUROC 0.95 internally and 0.96 externally; high-risk output predicted future dysfunction	CNN prediction of LV systolic dysfunction from ECG	49,158 training patients; 21,068 internal-test and 42,984 external-validation patients	Mayourian et al. [11]—AI-ECG/CHD

Abbreviations: AI, artificial intelligence; AUROC, area under the receiver operating characteristic curve; CHD, congenital heart disease; CNN, convolutional neural network; ECG, electrocardiography; HLHS, hypoplastic left heart syndrome; LV, left ventricular; ML, machine learning.

## 2. Artificial Intelligence Across the Diagnostic Pathway

The diagnostic evaluation of a child with a suspected inherited cardiovascular disorder requires integration of clinical presentation, family history, extracardiac manifestations, ECG and rhythm data, cardiovascular imaging, laboratory findings, and genomic information. AI-assisted systems may contribute to this process by structuring phenotypes, analyzing physiological signals and images, and identifying patients who warrant specialist or genomic assessment. Their value depends on whether performance persists across ages, institutions, acquisition protocols, and disease prevalence [1,12].

AI is therefore best conceptualized as an analytical layer across the diagnostic pathway rather than as a single diagnostic test. Tasks range from view classification and waveform analysis to multimodal models that combine clinical, imaging, electrophysiological, and molecular data. In pediatric cardiovascular genetics, the relevant benchmark is not technical accuracy alone but whether the system improves recognition, triage, or decision-making beyond established clinical workflows.

### 2.1. Computational Phenotyping and Electronic Health Records

Precise phenotypic characterization is fundamental to rare-disease diagnosis, yet clinically relevant information is often distributed across unstructured notes, imaging reports, discharge summaries, and records from multiple specialties. Natural-language processing and phenotype-mapping approaches can convert these data into structured concepts, including Human Phenotype Ontology terms, that support genomic prioritization and recognition of syndromic patterns [12].

AI-assisted phenotyping may therefore function as pre-genomic triage and as a post-sequencing aid to variant prioritization. However, missing data, copied text, inconsistent terminology, and center-specific documentation practices can systematically distort predictions; expert phenotype curation remains important, particularly when model output influences test selection or interpretation [12].

### 2.2. AI-Assisted Electrocardiography and Physiological Signals

The electrocardiogram is inexpensive, widely available, and central to inherited arrhythmias, cardiomyopathies, and congenital heart disease. Deep-learning models can analyze the full waveform rather than only predefined measurements and may detect patterns not readily apparent to visual inspection. Pediatric cardiovascular evidence is strongest when such models are evaluated in independent populations; large multicenter congenital-heart-disease studies now provide examples of externally validated AI-ECG performance, although these applications are not inherently genetics-specific [4,11,13].

### 2.3. Echocardiography

Echocardiography is the principal pediatric cardiac imaging modality but is highly operator- and protocol-dependent. Congenital heart disease adds further complexity because anatomy, age, prior interventions, equipment, and institutional acquisition practices vary substantially [5].

EchoFocus-CHD illustrates both the promise and fragility of this approach. The model detected critical and non-critical congenital heart defects with strong internal performance, but critical-CHD AUROC fell to 0.77 during external validation; retraining with more diverse data improved international-cohort performance to 0.87 [5]. This example illustrates that model transportability may depend as strongly on acquisition protocols, labeling practices, and population composition as on the underlying algorithm architecture.

In inherited cardiomyopathies, automated quantification of ventricular function, wall thickness, deformation, trabeculation, and atrial dimensions may improve reproducibility and longitudinal follow-up. Pediatric use nevertheless requires age- and body-size-aware reference ranges and evidence that subtle model-detected patterns add information beyond conventional imaging and genotype-aware specialist assessment [2].

### 2.4. Cardiovascular Magnetic Resonance, Computed Tomography, and Image–Genotype Integration

Cardiovascular magnetic resonance and computed tomography generate high-dimensional anatomical and tissue data suitable for automated segmentation, reconstruction, and radiomic analysis. These methods can reduce manual workload and may support serial assessment of complex anatomy, ventricular function, fibrosis, and vascular dimensions [14].

In pediatric congenital heart disease and heritable aortopathy, however, advanced-imaging cohorts remain relatively small and heterogeneous, particularly after surgery. Differences in scanners, sequences, reconstruction methods, and anatomy can limit transportability, so clinical use requires independent validation rather than extrapolation from adult or single-center imaging datasets [14].

### 2.5. Recognition of Syndromic and Multisystem Disease

Cardiovascular abnormalities may also be the presenting feature of a multisystem genetic syndrome. Computer-vision and phenotype-matching systems can support recognition of syndromic patterns from facial morphology and extracardiac findings, but these tools should be viewed as hypothesis-generating aids that require clinical genetic assessment and confirmation [15,16].

## 3. Artificial Intelligence in Cardiovascular Genomics

Genomic testing is integral to the evaluation of suspected inherited cardiovascular disease, but the principal bottleneck has shifted from data generation to clinically defensible interpretation. Panels, exome or genome sequencing, copy-number analysis, mitochondrial testing, and emerging multi-omics approaches can identify large numbers of candidate variants whose relevance depends on disease mechanism, inheritance, segregation, phenotype, and evidence quality [1] (Table 2).

Artificial intelligence and related computational approaches have been developed for multiple stages of genomic analysis, although many have not yet been specifically validated in pediatric cardiovascular genetics. Their roles include variant detection, effect prediction, phenotype-driven prioritization, multimodal interpretation, and reanalysis. Computational output should therefore be treated as supporting evidence within clinical variant-classification and disease-assessment frameworks rather than as an autonomous molecular diagnosis [17,18].

The importance of phenotype-rich genomic assessment was demonstrated by the Cardiac Genome Clinic, which applied genome sequencing to 111 families with pediatric heart disease and identified causative variants in 14 families (12.6%). Among these findings, seven variants were de novo and six were inherited from parents with no or only subclinical cardiac manifestations. A positive genomic result was associated with the presence of extracardiac features (*p* = 0.02), but not with a positive family history of cardiac lesions (*p* = 0.67), illustrating both the value of systematic multisystem phenotyping and the limitations of family history alone for selecting children for genomic testing [19].

### 3.1. AI-Assisted Variant Calling and Quality Control

The initial bioinformatic analysis of sequencing data requires accurate identification of sequence differences from the reference genome. DeepVariant is a general genomic deep-learning variant caller, not a pediatric cardiovascular-specific application; it learns sequencing-read features associated with single-nucleotide variants and small insertions or deletions and has demonstrated broad technical utility across sequencing contexts [17].

However, variant-calling accuracy depends on the sequencing technology, read length, coverage, alignment strategy, and genomic region under investigation. A high-performing genome-wide model does not eliminate the need to verify whether clinically relevant regions were adequately covered. Orthogonal confirmation may remain necessary, particularly for variants with major consequences for predictive testing, surveillance, or invasive treatment [17,18].

### 3.2. Missense Variant Effect Prediction

Missense variants are among the most frequent and difficult findings in cardiovascular genetic testing. AlphaMissense and related protein- or sequence-based models are general genomic tools rather than pediatric cardiovascular-specific systems. More recent disease-specific approaches such as DYNA fine-tune genomic foundation models using cardiovascular rare-variant data and have improved held-out variant-effect prediction, illustrating a potentially more clinically contextualized direction [6,20,21].

Computational evidence should be applied within established variant-classification frameworks and should not be counted repeatedly when predictors are correlated. Recent clinical-grade evaluation also underscores the need for caution: in a rare-disease cohort spanning 45 disorders, AlphaMissense showed substantial discordance with expert-curated pathogenic classifications, with reported precision of 32.9% and recall of 57.6% for pathogenic variants [22]. Predictions must therefore be integrated with population frequency, segregation, de novo occurrence, phenotype specificity, functional evidence, gene–disease validity, and known disease mechanism [18,20].

### 3.3. Prediction of Splicing Effects

The clinical effect of a genomic variant cannot always be inferred from its position relative to the canonical splice sites. Variants located within exons, deep intronic regions, or regulatory sequences may create or disrupt splice sites, alter branch points, activate cryptic exons, or modify splicing regulatory elements. These effects are particularly relevant to genome sequencing, which identifies large numbers of intronic variants that cannot be evaluated through coding consequence alone [23].

### 3.4. Structural Variants, Copy-Number Changes, and Complex Genomic Alterations

Copy-number and structural variants contribute to isolated and syndromic cardiovascular disease through dosage changes, gene disruption, regulatory effects, and complex rearrangements. Detection remains method-dependent, and short-read sequencing may miss repetitive, balanced, or otherwise complex alterations. Recent long-read genomic approaches broaden the range of detectable clinically relevant variation but still require standardized analytical and interpretive workflows [24].

Interpretation of complex variation remains limited by incomplete reference datasets and uneven representation of genomic diversity. Long-read sequencing, phasing, transcriptomic evidence, and functional studies may resolve selected cases, but technical novelty does not remove the need for segregation analysis and disease-specific biological interpretation [24].

### 3.5. Non-Coding Variation and Regulatory Effects

The majority of the human genome is non-coding, yet clinical interpretation remains concentrated on protein-coding exons and canonical splice sites. Genome sequencing can identify regulatory variants affecting promoters, enhancers, untranslated regions, chromatin organization, and non-coding RNAs, but determining which of these findings are functionally relevant is exceptionally difficult [25].

At present, non-coding predictions are most appropriately used to prioritize variants for additional investigation rather than to establish pathogenicity. Supporting evidence may include segregation, recurrence in similarly affected individuals, allele-specific expression, reporter assays, chromatin studies, or transcriptomic analysis. The interpretation of non-coding variation represents one of the areas in which AI has considerable potential but remains furthest from routine clinical implementation [18,25].

### 3.6. Phenotype-Driven Gene and Variant Prioritization

The diagnostic value of genomic data increases when variants are interpreted against a precisely defined phenotype. Computational phenotype-driven prioritization tools combine variant-level information with semantic similarity between patient features and known gene–disease associations. Exomiser, for example, integrates allele frequency, predicted pathogenicity, inheritance, human and model-organism phenotypes, and interaction data to rank candidate genes and variants [12,25].

Automated extraction of HPO terms may facilitate prioritization, but expert curation remains important. Hybrid systems are increasingly combining evidence-based scores, machine learning, phenotypic information, and language-model explanations; aiDIVA, published in 2026, is one recent example of this integrated direction [26]. The most clinically plausible workflow remains iterative: phenotype guides prioritization, candidate variants trigger reverse phenotyping, and revised clinical information is returned to the analysis [12,25,26].

### 3.7. Multimodal and Multi-Omics Integration

A genomic variant represents only one level of biological information. Transcriptomics, proteomics, metabolomics, epigenomics, and cellular models may provide evidence that a candidate variant alters gene expression, RNA processing, protein abundance, or downstream molecular pathways. AI methods are well suited to integrating these high-dimensional datasets and identifying patterns that cannot be detected through analysis of each modality in isolation [27].

Multimodal models may eventually combine genomic variation with ECG, echocardiographic, magnetic resonance, laboratory, and longitudinal clinical data. Such an approach could distinguish variants that are molecularly disruptive but clinically unrelated from those that produce a coherent disease-specific signature. However, multi-omics datasets are often small, incomplete, and generated using different laboratory platforms. Without careful validation, models may learn technical batch effects rather than biologically meaningful relationships [27].

### 3.8. Automated Reanalysis and the Dynamic Genome

A negative genomic result is not necessarily permanent. New gene–disease relationships, variant classifications, phenotypic annotations, and computational methods continue to evolve, while childhood phenotypes may become more specific with age. Automated reanalysis is therefore a particularly realistic near-term application: Talos recovered 90% of known diagnoses in validation and identified additional diagnoses in 5.1% of a large undiagnosed rare-disease cohort, demonstrating the feasibility of frequent, systematic reinterpretation at scale [7].

An ideal system would continuously monitor changes in genomic knowledge and alert the clinical team when a previously reported variant acquires new evidence or when a newly described gene matches the patient’s phenotype. However, this raises practical and ethical questions regarding the frequency of reanalysis, responsibility for reinterpretation, recontacting families, consent, and management of uncertainty [7].

**Table 2:** Principal AI-assisted tasks in cardiovascular genomic analysis.

Principal Limitation	Potential Clinical Contribution	AI-Assisted Task	Analytical Stage
Platform-, coverage-, and region-dependent performance	Improved detection consistency and prioritization of technically credible variants	Calling and quality assessment of SNVs and small indels	Variant detection
Predictions are supporting evidence and may be correlated	Prioritization of variants for ACMG/AMP evidence review	Assessment of missense, splice-altering, and regulatory variants	Variant-effect prediction
Reduced sensitivity in repetitive regions and for complex alleles	Recognition of dosage changes and disrupted cardiovascular genes	Detection of CNVs and complex genomic rearrangements	Structural-variant analysis
Dependent on phenotype quality and current knowledge bases	Ranking of candidate genes and support for reverse phenotyping	Integration of HPO terms, inheritance, and gene–disease knowledge	Phenotype-driven prioritization
Small datasets, missing modalities, and batch effects	Mechanistic assessment of candidate variants and pathways	Combination of genomic, transcriptomic, proteomic, and clinical data	Multi-omics integration
Unresolved responsibility for reanalysis, recontact, and consent	Identification of diagnoses missed at the initial analysis	Monitoring of new gene–disease and variant evidence	Automated reanalysis

Abbreviations: ACMG/AMP, American College of Medical Genetics and Genomics/Association for Molecular Pathology; AI, artificial intelligence; CNV, copy-number variant; HPO, Human Phenotype Ontology; SNV, single-nucleotide variant.

## 4. Disease-Specific Applications

The evidence base is uneven across inherited cardiovascular disease groups. Congenital heart disease has the largest pediatric AI literature, whereas cardiomyopathies, channelopathies, and aortopathies more often rely on small cohorts, adult-derived methods, or disease-specific clinical knowledge rather than externally validated pediatric AI systems. This section therefore emphasizes where primary evidence exists and explicitly identifies areas in which applications remain mainly conceptual.

### 4.1. Inherited Cardiomyopathies

Inherited cardiomyopathies are natural candidates for multimodal analysis because diagnosis and risk assessment integrate family history, ECG, rhythm monitoring, imaging, and genetics. In children, hypertrophic phenotypes may reflect sarcomeric disease or phenocopies such as RASopathies, glycogen-storage, lysosomal, mitochondrial, or other syndromic disorders, while dilated and arrhythmogenic phenotypes span diverse genetic and acquired mechanisms [2,28].

AI-assisted imaging may quantify morphology, deformation, fibrosis, trabeculation, and longitudinal change more consistently than isolated manual measurements, potentially helping distinguish etiologies or identify progression. However, robust pediatric studies that combine imaging AI with independently validated genotype-specific prediction remain limited; much of the current evidence is therefore best viewed as an emerging application rather than an established clinical tool [2,28].

Label leakage is a particular concern in genotype-aware models. Leakage can occur whenever model inputs contain information used directly or indirectly to define the outcome—for example, when genotype contributes to a diagnostic label and is then reused as a predictor of that same diagnosis. Development and validation datasets should therefore use independently defined outcomes and clearly distinguish prediction of current phenotype from prediction of future penetrance.

### 4.2. Cardiac Channelopathies and Inherited Arrhythmias

Inherited channelopathies include long QT syndrome, Brugada syndrome, catecholaminergic polymorphic ventricular tachycardia, short QT syndrome, and rarer primary arrhythmia syndromes. Disease expression is age-, genotype-, trigger-, and treatment-dependent, and contemporary pediatric consensus guidance increasingly emphasizes gene-aware diagnosis and management [29]. AI studies are more limited than in congenital heart disease; the deep-learning LQTS ECG literature illustrates diagnostic potential, but broad gene-specific pediatric risk models remain insufficiently validated [13,29].

Genotype–phenotype modeling may eventually integrate ECG, exercise or ambulatory monitoring, treatment response, and variants in genes such as KCNQ1, KCNH2, SCN5A, and RYR2. Family-level data partitioning should therefore be used, with related individuals assigned to the same training, validation, or test partition; otherwise, models may learn family-specific patterns and substantially overestimate generalizability [29].

### 4.3. Heritable Aortopathies

Heritable thoracic aortic disease includes syndromic and non-syndromic disorders in which risk depends on the affected gene, variant, family history, anatomical segment, body size, age, and additional clinical factors rather than aortic diameter alone [30].

Primary pediatric AI studies specific to heritable aortopathy remain sparse. Consequently, proposed applications—such as gene-aware modeling of aortic growth, branch-vessel abnormalities, or intervention thresholds—should be described as future targets rather than validated clinical tools. This lack of disease-specific evidence is itself an important gap in the field [30].

### 4.4. Congenital Heart Disease

Congenital heart disease is the most extensively studied pediatric cardiovascular domain for AI-assisted imaging and outcome prediction. Large pediatric ECG and echocardiography cohorts, perioperative models, longitudinal single-ventricle studies, and externally validated AI-ECG models provide a substantially stronger empirical base than exists for most inherited cardiomyopathies or aortopathies [4,5,8,9,11]. Its genetic architecture, however, remains heterogeneous, ranging from chromosomal and copy-number disorders to monogenic, de novo, oligogenic, and multifactorial mechanisms [31].

Importantly, strong performance for detecting or prognosticating CHD does not automatically translate into genomic utility. Models intended to support cardiovascular genetics should distinguish anatomical classification from syndromic recognition, genomic triage, and genotype-informed prognosis and should be validated across age groups, institutions, devices, and referral settings [4,5,31].

### 4.5. Syndromic and Metabolic Cardiovascular Disease

Genetic, metabolic, and neuromuscular disorders with cardiovascular involvement are individually rare but clinically important because etiological diagnosis may alter treatment and surveillance. AI-assisted systems could combine extracardiac phenotype, biomarkers, imaging, ECG, and genomics to identify phenocopies or treatable mechanisms, but disease-specific pediatric evidence is currently limited [1,28].

Longitudinal multimodal models may be particularly useful in disorders such as dystrophinopathies, laminopathies, mitochondrial disease, and metabolic cardiomyopathies, where cardiac risk evolves alongside systemic manifestations. At present, these applications remain more plausible as structured decision-support targets than as validated AI products [28].

### 4.6. Toward Disease-Aware Multimodal Models

Disease-specific models may perform well for narrow tasks, but pediatric cardiovascular genetics rarely presents as an isolated classification problem. Future systems will likely need to be both multimodal and disease-aware to provide clinically meaningful predictions.

Different clinical questions require different labels, datasets, and validation strategies. A model that detects ventricular hypertrophy should not be assumed to predict sarcomeric genotype or sudden-death risk, and a variant-effect predictor does not determine disease severity in the child carrying that variant.

The most clinically relevant direction is interpretable integration of longitudinal phenotype, ECG, imaging, family history, and genomics. Such systems should rank hypotheses, show which features support them, quantify uncertainty, and suggest confirmatory evaluation rather than present pattern recognition as a final diagnosis.

## 5. Risk Stratification and Prognosis

The diagnosis of an inherited cardiovascular disorder does not, by itself, determine an individual child’s prognosis. Pathogenic variants within the same gene—and sometimes the same variant within a family—may be associated with markedly different ages at onset, disease severity, and risks of arrhythmia, heart failure, or vascular complications. Conventional risk scores generally use a limited number of clinical variables measured at a single time point and may not adequately represent nonlinear interactions, longitudinal change, or the modifying effects of genotype [2,29].

Artificial intelligence may enable more individualized risk estimation by integrating longitudinal clinical observations, electrocardiographic signals, ambulatory monitoring, imaging, biomarkers, genomic findings, treatment exposure, and family history. Potential applications include prediction of malignant arrhythmias and sudden cardiac death, progression of cardiomyopathy, adverse outcomes after congenital heart surgery, need for mechanical circulatory support or transplantation, and post-transplant survival. The clinical value of these models, however, depends not only on statistical discrimination but also on calibration, interpretability, external validity, and evidence that their use improves clinical outcomes [2].

### 5.1. Prediction of Ventricular Arrhythmias and Sudden Cardiac Death

Risk stratification for sudden cardiac death is one of the most important and challenging tasks in inherited cardiovascular disease. Preventive interventions can be lifesaving but are also associated with procedural complications, inappropriate therapies, device malfunction, infection, and substantial psychosocial consequences. Accurate identification of high-risk children is therefore essential [2,29].

Genomic information may add prognostic value when particular genes, variant locations, or molecular mechanisms are associated with different risks. Nevertheless, the use of individual variants as predictive features is complicated by rarity, familial clustering, and uncertain penetrance. Models may memorize family-specific patterns rather than learn generalizable biological relationships. Related individuals must therefore be handled carefully during data partitioning, and gene- or mechanism-level representations may be more robust than individual variant identifiers [2,29].

### 5.2. Progression of Cardiomyopathy and Heart Failure

Children with inherited cardiomyopathy may follow markedly different clinical trajectories. Some remain asymptomatic with stable ventricular function, whereas others develop progressive heart failure, malignant arrhythmias, or a need for mechanical circulatory support and transplantation. Prediction based on a single baseline examination is limited because disease evolution is inherently longitudinal [2,28].

AI may support earlier recognition of deteriorating patients and more efficient timing of referral to advanced heart-failure services. However, overly frequent model alerts may lead to unnecessary escalation, while false reassurance may delay treatment. Prospective evaluation should therefore determine whether model-guided monitoring improves the timing of intervention without creating excessive testing or alarm burden.

### 5.3. Risk Prediction in Congenital Heart Disease

Children with congenital heart disease face heterogeneous risks that extend from the perioperative period through long-term follow-up. Outcome is influenced by anatomical complexity, surgical procedure, residual lesions, ventricular morphology, extracardiac abnormalities, genetic diagnosis, prematurity, nutritional status, organ function, and postoperative physiological trajectories [31].

In a cohort of approximately 23,000 children undergoing congenital heart surgery, machine-learning models were developed to predict low cardiac output syndrome, pneumonia, renal failure, and deep venous thrombosis. Reported test-set areas under the curve ranged from approximately 0.89 to 0.97, depending on the outcome. Explainability analysis identified mechanical ventilation duration as an important contributor across several complications. These findings demonstrate the capacity of AI to derive strong predictions from large perioperative datasets, but they also illustrate the risk of temporal leakage. Variables that occur after the beginning of a complication may improve prediction without providing sufficient time for preventive action [8].

For children with single-ventricle physiology, machine learning has also been investigated for prediction of longer-term transplant-free survival after staged palliation. Such models may integrate anatomy, surgical history, ventricular function, atrioventricular-valve regurgitation, catheterization data, growth, and clinical events. Long-term prediction remains challenging because treatment strategies and surgical outcomes change over time, producing temporal drift in model performance [9].

### 5.4. Advanced Heart Failure and Transplantation

AI may contribute to several stages of pediatric heart transplantation, including timing of listing, estimation of wait-list mortality, donor–recipient matching, prediction of early graft failure, and long-term survival. Registry datasets provide relatively large sample sizes for rare pediatric outcomes and have therefore been frequently used for model development [10].

In an analysis of 4150 pediatric heart-transplant recipients, machine-learning survival models modestly outperformed a conventional Cox proportional-hazards model for prediction of one- and three-year survival. Random survival forests achieved the highest reported concordance index of 0.68. This example is important because it demonstrates that more complex algorithms do not necessarily produce dramatic improvements. Transplant outcomes are influenced by many factors that may be incompletely captured in registries, and high-quality prediction may remain difficult regardless of model architecture [10].

### 5.5. Longitudinal and Dynamic Risk Assessment

Most cardiovascular risk tools generate predictions from variables measured at a single encounter, an especially restrictive approach in children because body size, anatomy, electrical characteristics, treatment, and disease expression change with development. Longitudinal pediatric studies in congenital heart disease show the value of updating risk estimates as new perioperative, ECG, imaging, and clinical information becomes available [9,11].

### 5.6. Evaluating Prognostic Models for Clinical Use

The area under the receiver operating characteristic curve is commonly reported but is insufficient to establish clinical usefulness. A model can distinguish higher-risk from lower-risk patients while systematically overestimating or underestimating absolute risk. Calibration is especially important when predictions are used to support preventive interventions [32].

The evaluation of prognostic AI systems should encompass discrimination and calibration; sensitivity, specificity, and predictive values at clinically relevant thresholds; time-dependent performance for survival outcomes; external validation across institutions and populations; comparison with established clinical models; decision-curve analysis and net clinical benefit; robustness to missing data and changes in clinical practice; subgroup performance across age, sex, ancestry, diagnosis, and genotype; and prospective assessment of their effects on clinical decision-making and patient outcomes. A practical evaluation framework is presented in Table 3.

PROBAST+AI provides a complementary framework for appraising methodological quality, risk of bias, and applicability across both regression-based and AI prediction models. It distinguishes model development, assessed with 16 targeted signalling questions, from model evaluation, assessed with 18 questions, while organizing both components into four domains: participants and data sources, predictors, outcome, and analysis. Fairness and algorithmic bias are embedded throughout the framework, making it particularly relevant to small, heterogeneous, and potentially unrepresentative pediatric datasets [33].

Explainability methods such as Shapley Additive Explanations can indicate which variables contributed to a prediction, but they do not establish causality. A feature may be predictive because it reflects early deterioration, clinician behavior, or access to care rather than disease mechanism [34]. Table 3 therefore addresses model-level evaluation before clinical use, whereas Table 4 addresses the distinct problem of deployment, governance, monitoring, and accountability once a model is considered for implementation.

### 5.7. From Risk Prediction to Clinical Action

A prognostic model has limited value unless its output is linked to a clearly defined clinical action. Potential applications include modification of surveillance intervals, additional rhythm monitoring, advanced imaging, referral to electrophysiology or heart-failure services, consideration of preventive treatment, or multidisciplinary reassessment. The intervention triggered by the prediction must itself have a favorable balance of benefit and harm [32].

AI-assisted risk stratification should ultimately operate as a dynamic partnership between computational prediction and specialist judgment. Genotype, phenotype, and longitudinal data can be integrated to produce individualized estimates, while clinicians assess biological plausibility, patient and family preferences, treatment options, and uncertainty. This model of augmented decision-making is more appropriate than autonomous prediction for rare and inherited cardiovascular disease in children.

## 6. Precision Therapeutics

Precision therapeutics aims to select or develop interventions according to the molecular mechanism, clinical phenotype, and predicted treatment response of an individual patient. In pediatric cardiovascular genetics, this concept is particularly relevant because similar cardiac phenotypes may arise from biologically distinct disorders. Conversely, pathogenic variants affecting the same gene may produce variable disease severity and therapeutic response. Artificial intelligence may support precision treatment through patient stratification, pharmacogenomic prediction, drug discovery and repurposing, identification of candidates for gene- or RNA-based therapies, and longitudinal assessment of therapeutic response [35,36].

### 6.1. Treatment Selection and Response Prediction

Most conventional treatment recommendations are derived from population-level evidence and may not fully account for the heterogeneity of rare pediatric cardiovascular disorders. AI models can integrate genotype, age, imaging, electrophysiology, biomarkers, comorbidities, and previous treatment response to identify clinically relevant patient subgroups.

Prediction of response must be distinguished from prediction of prognosis. A patient at high risk of progression is not necessarily the patient most likely to benefit from a particular intervention. Reliable treatment-selection models require data from treated and untreated patients, careful adjustment for confounding by indication, and preferably randomized or prospectively collected evidence [32].

### 6.2. Pharmacogenomics and Medication Safety

Pharmacogenomics investigates how inherited variation influences drug metabolism, efficacy, and toxicity. In cardiovascular medicine, relevant examples include variation affecting drug-metabolizing enzymes, transporters, receptors, and ion channels. AI may integrate pharmacogenomic findings with age, organ function, concurrent medication, ECG characteristics, and clinical response to predict individualized treatment effects [35].

Pediatric pharmacogenomic models face additional limitations related to developmental pharmacology. Drug absorption, distribution, metabolism, and elimination change throughout childhood, and models derived from adults may not be transferable to neonates, infants, or adolescents. Pediatric-specific data and age-aware modeling are therefore essential [35].

### 6.3. AI-Assisted Drug Discovery and Repurposing

Traditional drug development is costly and inefficient, particularly for rare diseases with small potential trial populations. AI can analyze molecular structures, protein interactions, biological networks, multi-omics data, and existing drug-response datasets to prioritize compounds and therapeutic targets [36].

In cardiovascular disease, AI-assisted drug discovery may identify molecules predicted to modify pathways involved in sarcomeric function, calcium handling, fibrosis, mitochondrial dysfunction, vascular signaling, or ion-channel activity. Generative models may propose new molecular structures, while protein-structure models can support prediction of binding sites and drug–target interactions [36].

Drug repurposing is especially attractive for rare pediatric disorders because it evaluates medications with existing pharmacological and safety information. Machine-learning models can compare disease-associated molecular signatures with drug-induced expression profiles or predict interactions between approved drugs and candidate targets. For example, AI-assisted screening has been used to identify previously unrecognized interactions between approved compounds and cardiac calcium-handling proteins [36].

Computational prioritization remains hypothesis-generating. Predicted compounds require biochemical validation, disease-relevant cellular testing, appropriate animal models, and clinical investigation. Models may identify statistical similarities that do not translate into therapeutic effects, while apparent efficacy in adult or non-cardiac datasets may not predict safety in children [36].

### 6.4. Gene- and RNA-Based Therapies

Gene replacement, genome editing, allele-specific silencing, exon modification, and antisense oligonucleotide therapies offer the possibility of targeting the primary cause of inherited cardiovascular disease. Potential indications include monogenic cardiomyopathies, channelopathies, neuromuscular disorders with cardiac involvement, and selected metabolic diseases [37].

For variants affecting RNA splicing, deep-learning predictors may identify the abnormal splice event and support the design of antisense strategies. Similarly, models of protein structure and sequence conservation may help select variants for editing or allele-specific silencing. These applications demonstrate a potential transition from AI-assisted variant interpretation to AI-assisted therapeutic design [23,37].

### 6.5. Patient Selection for Molecular Therapy

Rare-disease trials frequently include small and heterogeneous populations. AI may assist in identifying patients who share a molecular mechanism, disease stage, or biomarker profile and are therefore most likely to benefit from a targeted intervention [37].

Multimodal selection may be superior to genotype alone. Two patients carrying variants in the same gene may differ in residual protein function, myocardial fibrosis, disease stage, or reversibility. Integration of genomic findings with transcriptomic data, imaging, biomarkers, and longitudinal progression may distinguish patients with active and potentially modifiable disease from those with irreversible structural damage.

However, algorithmic selection for experimental therapy raises significant ethical concerns. Underrepresentation of particular ancestries or healthcare systems could systematically reduce access for groups poorly represented in training datasets. AI should support, rather than replace, transparent eligibility criteria and multidisciplinary review [38].

### 6.6. Toward AI-Assisted Precision Cardiovascular Care

The long-term objective is a learning system in which diagnosis, treatment, and longitudinal outcome continuously inform one another. Following molecular diagnosis, an AI-assisted platform could identify relevant therapies or trials, estimate treatment response, recommend biomarkers for monitoring, and update predictions as new clinical data emerge.

The most realistic near-term role of AI is therefore not autonomous prescribing but the prioritization of therapeutic hypotheses and support of multidisciplinary decisions. By connecting genotype, molecular mechanism, clinical phenotype, and longitudinal response, AI may help transform genomic diagnosis from an endpoint into a pathway toward individualized treatment.

## 7. Challenges and Requirements for Clinical Translation

Despite rapid methodological progress, most AI systems in pediatric cardiovascular genetics remain at the development or retrospective validation stage. Translation into clinical practice is limited by the rarity and heterogeneity of the underlying diseases, variable data quality, insufficient external validation, limited interpretability, privacy concerns, and uncertainty regarding regulatory responsibility. These challenges are amplified in children because physiology and disease expression change with age and because inaccurate predictions may influence lifelong surveillance and treatment [38,39].

### 7.1. Small, Heterogeneous, and Imbalanced Datasets

Rare inherited cardiovascular disorders are represented by small patient cohorts, often assembled across long periods during which diagnostic criteria, sequencing technologies, imaging protocols, and treatment strategies have changed. Individual diagnostic categories may contain only a small number of clinically relevant events, limiting the development of reliable prognostic models [39].

### 7.2. Population Representation and Algorithmic Bias

Genomic databases and cardiovascular cohorts disproportionately represent individuals of European ancestry and patients treated at large academic centers. Variant-frequency estimates, pathogenicity predictions, polygenic models, and AI-assisted clinical tools may therefore perform less accurately in underrepresented populations [38].

### 7.3. External Validation, Domain Shift, and Model Drift

Random division of a single institutional dataset into training and test subsets provides only limited evidence of generalizability. Patients from the same institution may share equipment, documentation practices, referral patterns, and clinical decision-making. External validation in independent institutions and populations is therefore essential [5,39].

### 7.4. Interpretability and Clinical Plausibility

Complex models may generate accurate predictions without providing a clinically understandable explanation. In high-stakes cardiovascular decisions, clinicians must be able to determine which data contributed to the result and whether the output is biologically plausible [34].

Interpretability is particularly important when genomic and phenotypic predictions are combined. The system should distinguish evidence supporting the molecular diagnosis from evidence supporting clinical risk and should communicate uncertainty at both levels.

### 7.5. Privacy, Consent, and Data Governance

Genomic data are identifiable, persistent, and informative about biological relatives. Their combination with facial images, pedigrees, longitudinal health records, and cardiovascular imaging increases re-identification risk. Pediatric consent adds a developmental dimension because parents or guardians initially authorize data use, while the child may later acquire capacity for independent preferences and reassent [40].

Data-sharing frameworks should define access, secondary uses, retention, commercial involvement, recontact, and the handling of changing preferences. Dynamic-consent platforms offer one possible mechanism for ongoing communication, granular choices, and reassent as children mature; notably, such an approach has been evaluated in a cardiovascular genetic-disorders cohort, although uptake and implementation remain practical challenges [41]. Federated learning may reduce the need to centralize raw data and has recently been studied directly for multi-site genetic-variant pathogenicity annotation, but it does not eliminate governance, security, or bias concerns [42].

### 7.6. Automation Bias and Clinical Responsibility

Clinicians may place excessive confidence in algorithmic recommendations, particularly when outputs are presented as precise probabilities. Conversely, poorly integrated tools may be ignored even when they provide useful information. AI systems should therefore be designed around actual clinical workflows and should clearly communicate uncertainty and situations in which the input falls outside the model’s validated domain [38].

Responsibility for clinical decisions must remain explicit. A model may prioritize a variant, suggest a diagnosis, or estimate risk, but the final interpretation requires consideration of evidence not available to the algorithm. Human oversight is essential when predictions influence cascade testing, surveillance of asymptomatic children, device implantation, transplantation, or access to molecular therapy [38].

### 7.7. Demonstrating Clinical Utility

Strong retrospective performance does not establish that an AI system improves care. Clinical utility requires evidence that model use changes decisions appropriately, shortens time to diagnosis, improves outcomes, or reduces unnecessary testing without disproportionate harm [43].

Transparent reporting and staged clinical evaluation are necessary for reproducibility and safe translation. SPIRIT-AI and CONSORT-AI provide reporting guidance for protocols and trials involving AI interventions, while recent implementation frameworks emphasize sequential assessment of safety, efficacy, effectiveness relative to existing care, and post-deployment monitoring [43,44,45].

### 7.8. Requirements for Responsible Implementation

Operational governance frameworks increasingly translate high-level principles into health-system processes. FAIR-AI, for example, combines structured pre-implementation review, risk categorization, safe implementation planning, end-user transparency, and post-implementation monitoring [46]. Together with model-quality frameworks such as PROBAST+AI, such approaches reinforce that trustworthy deployment requires evaluation of data quality, fairness, interpretability, clinical relevance, workflow integration, accountability, and ongoing performance rather than accuracy alone [33,46].

Clinical translation requires more than an accurate algorithm; a deployable system should have a clearly defined clinical purpose and intended population, standardized and quality-controlled inputs, independent multicenter external validation, calibrated predictions accompanied by uncertainty estimates, interpretable outputs with access to supporting evidence, documented performance across relevant subgroups, prospective evaluation of clinical benefit, secure data governance and age-appropriate consent procedures, continuous monitoring for model drift and adverse effects, and multidisciplinary oversight with a mechanism for clinician override. The corresponding requirements for responsible deployment are summarized in Table 4.

The transition from experimental models to clinical decision support should therefore be gradual and evidence-based. In pediatric cardiovascular genetics, the objective is not maximum automation but reliable augmentation of specialist care.

## 8. Future Directions

The next phase of artificial intelligence in pediatric cardiovascular genetics will likely move from single-task models toward multimodal systems capable of integrating clinical, electrophysiological, imaging, and molecular information. Such integration more closely reflects clinical reasoning, in which no individual data source provides a complete representation of an inherited cardiovascular disorder [11].

Foundation models trained on large amounts of unlabeled or weakly labeled data represent one promising direction. Through self-supervised learning, these models can acquire reusable representations of ECGs, imaging studies, genomic sequences, or clinical text and subsequently be adapted to rare disorders using relatively small labeled datasets. This may be particularly valuable in pediatric cardiovascular genetics, where expertly annotated cohorts are limited. Recent ECG deep-learning models have demonstrated improved performance in low-data settings and across several diagnostic tasks. Multimodal models combining ECG and cardiovascular imaging have similarly shown that complementary data sources may improve prediction beyond either modality alone [11,47].

Future systems should also incorporate longitudinal development. Pediatric models must account for changes in body size, cardiovascular physiology, phenotype, treatment, and disease penetrance over time. Rather than generating a fixed diagnosis or risk estimate, they should update predictions as new clinical, imaging, and molecular data become available. This could support personalized surveillance of genotype-positive children and earlier recognition of disease progression [11].

Digital twins represent a more ambitious extension of individualized modeling. A cardiovascular digital twin is a patient-specific computational representation updated using anatomical, physiological, and potentially genomic data. In principle, such models could simulate disease progression or compare expected responses to different interventions. In children with congenital or inherited cardiovascular disease, digital twins might support procedural planning, longitudinal prediction, or evaluation of molecular therapies. At present, however, most applications remain conceptual or experimental and require prospective evidence before clinical implementation [48].

The most important advance will not necessarily be the development of larger models, but the creation of clinically useful systems evaluated in representative pediatric populations. Future research should prioritize shared data standards, prospective multicenter studies, transparent reporting, and direct comparison with existing clinical workflows. Children and families should also be involved in decisions regarding consent, acceptable data use, communication of uncertainty, and the role of AI in their care [38,43].

Ultimately, successful AI systems should function as components of a learning healthcare network. Genomic diagnosis, longitudinal phenotype, treatment, and outcome would continuously contribute to improved models, while updated predictions would remain subject to multidisciplinary clinical review. This approach offers a realistic pathway from isolated algorithmic demonstrations toward equitable and evidence-based precision care.

## 9. Conclusions

The most defensible current role of AI in pediatric cardiovascular genetics is as an assistive layer that links complex phenotype and cardiovascular data with genomic interpretation while making uncertainty explicit. Evidence has progressed furthest for selected ECG, echocardiographic, and outcome-prediction applications; by contrast, many genomic tools remain general-purpose methods, and truly pediatric cardiovascular genotype-aware systems are only beginning to emerge.

The central opportunity is therefore not a single high-performing algorithm but a longitudinal, multimodal framework in which phenotype, family history, ECG, imaging, genomic findings, and molecular data are interpreted together. Such systems may improve diagnosis, surveillance, and therapeutic prioritization only if they add value beyond specialist workflows and remain robust across institutions and populations.

Current evidence nevertheless remains dominated by retrospective studies, selectively assembled cohorts, and models without independent pediatric validation. Population imbalance, familial clustering, age-dependent disease expression, domain shift, privacy concerns, and incomplete calibration or interpretability continue to constrain translation [5,38,39].

Accordingly, the question is no longer whether AI can produce strong retrospective performance, but which applications can demonstrate external validity, calibrated and clinically useful predictions, prospective benefit, and responsible governance. Pediatric cardiovascular genetics provides a particularly demanding test case because errors may affect lifelong surveillance, cascade testing, invasive treatment, and access to molecular therapies.

With responsible development and multidisciplinary oversight, AI has the potential to augment the expertise of pediatric cardiologists, clinical geneticists, imaging specialists, molecular diagnosticians, and bioinformaticians. Its most valuable contribution may be a transparent and continuously evaluated decision-support framework that connects deep phenotyping, cardiovascular data, genomics, risk prediction, and precision therapeutics without displacing specialist judgment.

## Figures and Tables

**Table 3:** Core domains for evaluating prognostic AI systems in pediatric cardiovascular genetics.

Clinical Relevance	Recommended Assessment	Evaluation Domain
Determines whether predicted risks are accurate and actionable	Discrimination, calibration, sensitivity, specificity, and predictive values at clinically relevant thresholds	Predictive performance
Accounts for censoring and changing risk over follow-up	Time-dependent discrimination and calibration for survival outcomes	Time-to-event performance
Tests robustness to differences in patients, equipment, and clinical practice	Independent external validation across institutions, populations, and time periods	Generalizability
Quantifies incremental value and net clinical benefit	Comparison with established clinical models and decision-curve analysis	Comparative value
Identifies conditions under which performance may deteriorate	Sensitivity analyses for missing data, model drift, and changes in practice	Robustness
Detects clinically important performance disparities	Subgroup performance across age, sex, ancestry, diagnosis, and genotype	Equity
Establishes benefit beyond retrospective statistical accuracy	Prospective assessment of effects on decisions, workflow, and patient outcomes	Clinical utility

Abbreviation: AI, artificial intelligence.

**Table 4:** Requirements for responsible clinical implementation of AI systems.

Operational Safeguard	Minimum Requirement	Implementation Domain
Use restricted to the validated indication and population	Clearly defined purpose, intended population, and decision supported	Clinical scope
Automated quality checks and rejection of invalid inputs	Standardized acquisition and quality-controlled clinical, imaging, and genomic data	Input quality
Warnings for out-of-domain cases and low-confidence outputs	Independent multicenter validation, calibrated predictions, and uncertainty estimates	Validation and uncertainty
Specialist review of biological plausibility and actionability	Clinically understandable outputs linked to supporting evidence	Interpretability
Ongoing disparity monitoring and predefined mitigation procedures	Documented performance across relevant demographic and disease subgroups	Equity
Audit trails, data minimization, and reassessment of consent over time	Secure data handling, defined access, and age-appropriate consent	Governance and consent
Version control, periodic reassessment, and incident reporting	Continuous surveillance for drift, errors, and adverse effects	Monitoring
Clinician override and escalation pathways for contested outputs	Multidisciplinary oversight with explicit accountability	Clinical responsibility

Abbreviation: AI, artificial intelligence.

## Data Availability

No new data were created or analyzed in this study.
